# Use of commercially available wearable devices for physical rehabilitation in healthcare: a systematic review

**DOI:** 10.1136/bmjopen-2024-084086

**Published:** 2024-11-07

**Authors:** Ahmed Latif, Hasaneen Fathy Al Janabi, Meera Joshi, Gianpaolo Fusari, Leila Shepherd, Ara Darzi, Daniel R Leff

**Affiliations:** 1 Department of Surgery and Cancer, Imperial College London, London, UK; 2 University Hospitals Birmingham NHS Foundation Trust, Birmingham, Birmingham, UK; 3 Division of Surgery, Imperial College Healthcare NHS Trust, London, UK; 4 The Helix Centre, Imperial College London, London, UK

**Keywords:** REHABILITATION MEDICINE, Self-Management, Physical Therapy Modalities

## Abstract

**Abstract:**

**Objectives:**

To evaluate whether commercially available ‘off-the-shelf’ wearable technology can improve patient rehabilitation outcomes, and to categorise all wearables currently being used to augment rehabilitation, including the disciplines and conditions under investigation.

**Design:**

Systematic review following the Preferred Reporting Items for Systematic Reviews and Meta-Analysis 2020 statement checklist, and using the Grading of Recommendations, Assessment, Development and Evaluation approach.

**Data sources:**

Embase, MEDLINE, Web of Science and the Cochrane Library were searched up to and including July 2023.

**Eligibility criteria:**

We included trials and observational studies evaluating the use of consumer-grade wearables, in real patient cohorts, to aid physical therapy or rehabilitation. Only studies investigating rehabilitation of acute events with defined recovery affecting adult patients were included.

**Data extraction and synthesis:**

Two independent reviewers used a standardised protocol to search, screen and extract data from the included studies. Risk of bias was assessed using the Cochrane Methods Risk of Bias in Randomised Trials V.2 and Risk of Bias in Non-Randomised Studies of Interventions tools for randomised controlled trials (RCTs) and observational studies, respectively.

**Results:**

Eighteen studies encompassing 1754 patients met eligibility criteria, including six RCTs, six quasi-experimental studies and six observational studies. Eight studies used wearables in Orthopaedics, seven in Stroke Medicine, two in Oncology and one in General Surgery. All six RCTs demonstrated that wearable-driven feedback increases physical activity. Step count was the most common measure of physical activity. Two RCTs in orthopaedics demonstrated non-inferiority of wearable self-directed rehabilitation compared with traditional physiotherapy, highlighting the potential of wearables as alternatives to traditional physiotherapy. All 12 non-randomised studies demonstrated the feasibility and acceptability of wearable-driven self-directed rehabilitation.

**Conclusion:**

This review demonstrates that consumer-grade wearables can be used as adjuncts to traditional physiotherapy, and potentially as alternatives for self-directed rehabilitation of non-chronic conditions. Better designed studies, and larger RCTs, with a focus on economic evaluations are needed before a case can be made for their widespread adoption in healthcare settings.

**PROSPERO registration ID:**

CRD42023459567

**STRENGTHS AND LIMITATIONS OF THIS STUDY**

- This systematic review used a comprehensive search of all randomised trials and observational studies using consumer-grade wearables for rehabilitation of non-chronic conditions in healthcare, which has not been undertaken in the literature previously.
- The review used the Grading of Recommendations, Assessment, Development and Evaluation approach to evaluate the strength of evidence, and used the Cochrane Methods Risk of Bias in Randomised Trials V.2 and Risk of Bias in Non-Randomised Studies of Interventions tools to evaluate the risk of bias in all included studies.

## Introduction

 Physiotherapy remains the cornerstone for rehabilitation, particularly following key events such as undergoing surgery or suffering a cerebrovascular accident. Disability can profoundly impact patients’ quality of life, as well as have wider societal implications, including the financial burden of unemployment and community care.^1^ The benefits of physiotherapy are well documented across various medical and surgical disciplines.^2^,^9^ Despite the introduction of patient direct access pathways to physiotherapy in the UK, limited resources, including a shortfall of physiotherapists and long waiting times, remain widespread.^10^ Mobile health (mHealth) and the use of wearable technologies present a potential solution in addressing resource and staffing constraints, by facilitating self-directed rehabilitation. Technology-driven self-care has gained significant traction in modern day physiotherapy, however challenges remain to its widespread adoption, including a lack of infrastructure and funding.^11^

Over the past two decades, mHealth continues to gather interest, and has seen a rapid rise in usage globally.^12^,^14^ The introduction of flagship devices, including the iPhone in 2007, catalysed an arms race of smartphone technologies, with improving features and sensors annually.^15^ Coupled with high-speed internet consumer-grade devices have empowered consumers with technologies unimaginable 20 years ago.^16 17^ As of 2023, 94% of the UK’s adult population owns smartphones, with an estimated increase to over 60 million people in 2028.^18 19^ Likewise, there has been a steady rise in using activity-tracking wristwear.^20^ These technologies have empowered patients and healthcare providers to connect and share data seamlessly.^21^ mHealth applications are now capable of monitoring heart rate, temperature, blood pressure, sleep cycles, step count, calorie expenditure and oxygen saturation, enabling remote monitoring of physiological parameters.^22^,^25^ The COVID-19 pandemic accelerated the acceptability and use of mHealth worldwide, with a 38-fold increase compared with the pre-COVID-19 baseline.^26^ The use of telemedicine during the pandemic highlighted numerous advantages, including decreasing healthcare infrastructure burdens and increasing access.^27^,^29^

To date, mHealth in physiotherapy has largely focused on the development of biosensors that track patient movement.^30^ An example is shoe-based sensors,^31 32^ which incorporate vibrotactile feedback.^33 34^ Another example is smart garments which use inertial measurement units embedded in clothing.^35 36^ The utilisation of these technologies has largely been confined to research settings with limited clinical uptake due to manufacturing challenges, durability and affordability.^37^ Undoubtedly, using sensors of consumer-grade wearables provides a viable and cost-effective alternative.^30^ There has been a considerable rise in the use of consumer-grade wearables for measuring physical activity in research.^38^

Currently, there is no unified consensus among healthcare disciplines on the use of wearables, how they could be incorporated into current physiotherapy regimens and whose role it would be to monitor them. A range of devices have been explored across different healthcare settings though, to date, no cross-discipline review has been conducted of the devices being used for rehabilitation, or whether they impact patient outcomes. The primary aim of this review is to evaluate whether consumer-grade ‘off-the-shelf’ wearables can improve patient rehabilitation. We aim to review this within an adult patient population, requiring acute rehabilitation for non-chronic conditions. The secondary aim is to systematically identify and categorise wearables currently being used in rehabilitation, and the medical disciplines and conditions for which they are being used.

## Methods

This systematic review was conducted in accordance with the Preferred Reporting Items for Systematic Reviews and Meta-Analyses statement. The protocol has been registered in the International Prospective Register of Systematic Reviews, PROSPERO (Registration ID CRD42023459567). We used the recommended Grading of Recommendations, Assessment, Development and Evaluation (GRADE)^39^ tool to summarise findings.

### Literature search strategy

A systematic search of the Embase, MEDLINE, Cochrane Library and Web of Science databases was performed up to and including 31 July 2023, for studies reporting the use of consumer-grade wearables in physical rehabilitation. The search terms spanned two domains namely wearable technology and physiotherapy. The full search strategy was formulated with support from a Medical Librarian for Embase, and adopted for the remaining databases (online supplemental appendix A). Reference lists of retrieved full-text articles were also used to hand search and identify further relevant articles.

### Inclusion and exclusion criteria

Studies using consumer-grade wearables, tested on real patients to aid physical rehabilitation, were included. Only studies investigating rehabilitation of acute events with defined recovery affecting adult patients were included (encompassing surgery, musculoskeletal injury, stroke and undergoing cancer treatment). Studies investigating patients under 18 years of age or requiring rehabilitation for chronic conditions were excluded (eg, chronic obstructive pulmonary disease, ischaemic heart disease). Design concepts, case reports, unpublished data from conference abstracts, review articles and studies not in English were also excluded.

### Screening and data extraction

The online Covidence platform (Covidence systematic review software, Veritas Health Innovation, Australia; available at www.covidence.org) was used to import search results, remove duplicates, and for abstract and full-text screening. A standardised protocol was followed using the inclusion and exclusion criteria. Screening and data extraction were performed by two authors (AL and HA) and discussion among all authors was used to reach an agreement on any discrepancies. The following information was obtained from each study: first author, publication year, study country, study design, number of participants, duration of intervention, medical discipline, condition treated, type of device and where it is worn, device brand and model, operating system, device outcome measure, if device was used as an intervention or for monitoring, and the main study findings and limitations. Given the heterogeneity of study populations, design, recorded outcomes, wearables tested and conditions treated, a meta-analysis was not possible.

### Risk of bias assessment

A risk of bias assessment was independently performed by two authors (AL and HA), and discrepancies encountered were discussed to reach a consensus. The Cochrane Methods Risk of Bias in Non-Randomised Studies of Interventions (ROBINS-I) was used to assess the risk of bias in observational cohort and non-randomised experimental studies.^40^ This tool classifies bias into low-risk, moderate-risk, serious-risk and critical-risk across seven domains, namely confounding, selection of participants, classification of interventions, deviations from intended interventions, missing data, measurement of outcomes and selection of the reported results. For randomised controlled trials (RCTs), we used the Cochrane Methods Risk of Bias in Randomised Trials V.2 (RoB 2).^41^ This tool categorises the risk of bias into low-risk, some concerns or high-risk across five domains, namely randomisation process, deviation from the intended intervention, missing outcomes, measurement of the outcomes and selection of reported results.

### Patient and public involvement

Several of the authors use consumer-grade wearables daily. Though patients and the public were not involved in the design or synthesis of this review, patient and public feedback using surveys and group discussions has been sought on the use of wearable technology for rehabilitation, and play a role in the overall recommendations informed by this review.

## Results

**Figure 1:**
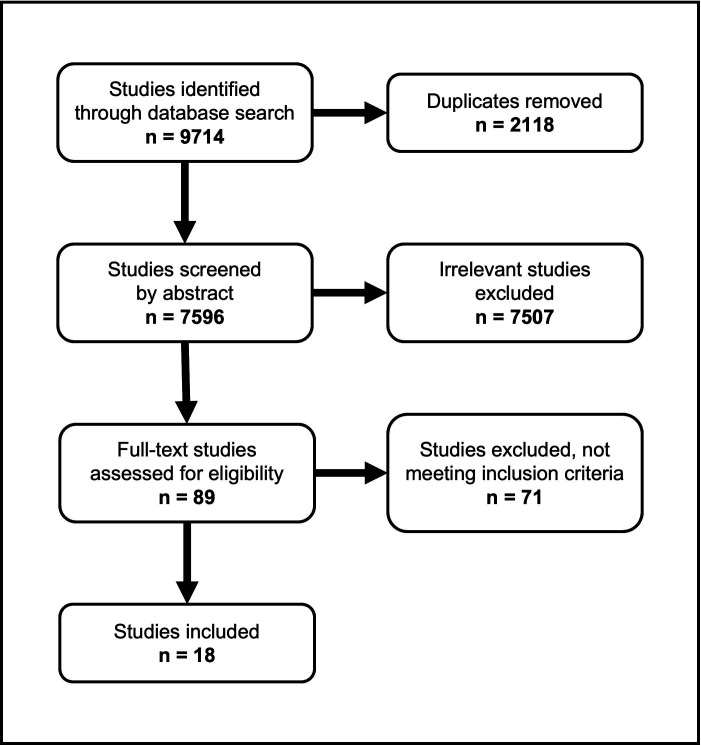
Identification of eligible studies.

In total, 7596 unique eligible studies were screened by abstract, of which 89 full-text studies were assessed for eligibility after exclusion (figure 1). Most studies excluded by abstract were due to descriptions of design concepts or using non-commercially available wearable devices including exoskeletons, without clinical outcome data. Of the 89 full-text studies assessed, 18 studies including data on 1754 patients meeting the eligibility criteria and were included in the final review.

### Characteristics of included studies

Six studies were RCTs,^42^,^47^ and 12 were non-randomised feasibility or prospective cohort studies.^48^,^59^ Table 1 summarises the study characteristics. All included studies took place between 2016 and 2022, reflecting the recent adoption of consumer-grade wearables in physical rehabilitation.

**Table 1:** Characteristics of included studies

First author	Country	Year of publication	Discipline	Index event(s) treated	Study design	Total (N)
Lawrie^44^	China and the UK	2018	Stroke Medicine	Stroke	RCT	24
Van der Walt^43^	Australia	2018	Orthopaedics	THA and TKA	RCT	163
Crawford^42^	USA	2021	Orthopaedics	TKA and PKA	RCT	452
Tripuraneni^46^	USA	2021	Orthopaedics	TKA	RCT	337
Kanai^47^	Japan	2018	Stroke Medicine	Stroke	RCT	48
Wolk^45^	Germany	2018	General Surgery	Major abdominal visceral surgery	RCT	132
Lyman^50^	USA	2020	Orthopaedics	THA and TKA	Observational	267
Broderick^52^	Ireland	2022	Oncology	Curative chemotherapy or radiotherapy	Quasi-experimental	45
Chae^51^	South Korea	2020	Stroke Medicine	Stroke	Quasi-experimental	23
Cheong^55^	South Korea	2018	Oncology	Postcancer chemotherapy	Quasi-experimental	75
Patterson^56^	USA	2020	Orthopaedics	THA and TKA	Observational	20
Christiansen^58^	USA	2020	Orthopaedics	TKA	Quasi-experimental	43
Paul^53^	The UK	2016	Stroke Medicine	Stroke	Quasi-experimental	23
Fusari^59^	The UK	2022	Stroke Medicine	Stroke	Quasi-experimental	12
Katzan^57^	USA	2021	Stroke Medicine	Stroke	Observational	15
Klassen^48^	Canada	2017	Stroke Medicine	Stroke	Observational	21
Mackenzie^54^	The UK and Australia	2022	Orthopaedics	Frozen shoulder	Observational	21
Toogood^49^	USA	2016	Orthopaedics	THA	Observational	33

PKApartial knee arthroplastyRCTrandomised controlled trialTHAtotal hip arthroplastyTKAtotal knee arthroplasty

### Medical disciplines

Nine studies used wearable technology in medical disciplines, seven of which were in stroke medicine^44 47 48 51 53 57 59^ and two in oncology.^52 55^ Of the nine surgical discipline studies, eight were in orthopaedics^42 43 46 49 50 54 56 58^ and one in general surgery.^45^ Of the RCTs, three were in Orthopaedics, two in Stroke Medicine and one in General Surgery. There were no RCTs in the field of Oncology. Table 1 includes a summary of the disciplines and events treated across all studies. All studies in stroke medicine used wearables for the rehabilitation of patients suffering from mild to moderate stroke. Of the two studies in oncology, Cheong *et al*^55^ tested wearable technology in the rehabilitation of patients undergoing chemotherapy for colorectal cancer. In the second oncology study by Broderick *et al*,^52^ researchers tested wearables in patients with cancer having completed chemotherapy or radiotherapy. Within orthopaedics, one study by Mackenzie *et al*^54^ investigated the use of wrist-worn tracker for frozen shoulder rehabilitation. The remaining seven Orthopaedics studies, all investigated the use of wearables in postoperative rehabilitation following lower limb arthroplasty surgery.^42 43 46 49 50 56 58^ The final study in General Surgery^45^ evaluated the use of wrist-worn trackers for postoperative rehabilitation after major abdominal visceral surgery.

### Wearable technologies, operating systems and outcome measures

Seven studies used apps on wearable smart devices,^42 44 46 50 51 53 59^ and 11 used wrist-worn activity trackers.^4345 47^,^49 52 54^ Step count was the most commonly used outcome measure of physical activity. A summary of the wearable technologies used and outcomes assessed is given in table 2.

**Table 2:** Characteristics of wearable technologies used in included studies

Study	Technology	Operating system	App or device	Device model	Outcomes measured	Measurement sensor	Real-time therapist monitoring?	Location of device tested
**RCTs**								
Lawrie *et al*^44^	App on smartwatch	Android	Unnamed app used on Android smartwatch	ZGPAX S8 smartwatch	Overall body movement	Smartwatch accelerometer	No	Wrist
Van der Walt *et al*^43^	Activity tracker	Garmin OS, Android or iOS	Garmin wristband	Vivofit 2	Step count	Wristband accelerometer	No	Wrist
Crawford *et al*^42^	App on smartphone and smartwatch	iOS and Apple watch OS	MyMobility app on iPhone and Apple watch	Not specified	Step count, flights of stairs, stand hours and heart rate	Smartwatch accelerometer, gyroscope and heart rate sensor	Yes	Wrist
Tripuraneni *et al*^46^	App on smartphone and smartwatch	iOS and Apple watch OS	MyMobility App on iPhone and Apple watch	Not specified	Step count, flights of stairs, stand hours and heart rate	Smartwatch accelerometer, gyroscope and heart rate sensor	Yes	Wrist
Kanai *et al*^47^	Activity tracker	Fitbit OS, Android or iOS	Fitbit wristband	Fitbit One	Step count	Wristband accelerometer	No	Waist belt
Wolk *et al*^45^	Activity tracker	Android, iOS or Huawei OS	Polar wristband	Loop activity tracker	Step count	Wristband accelerometer	No	Wrist
**Non-randomised quasi-experimental and observational studies**								
Lyman *et al*^50^	App on smartphone	Android or iOS	Moves App used on Android or iOS smartphones	N/A	Step count	Smartphone accelerometer	Yes	Pocket
Broderick *et al*^52^	Activity tracker	Fitbit OS, Android or iOS	Fitbit wristband	Fitbit One or Fitbit Flex	Step count	Wristband accelerometer	No	Wrist
Chae *et al*^51^	App on smartphone and smartwatch	Android	Unnamed app used on Android smartphone and smartwatch	LG W270 smartwatch	Limb movement	Smartwatch accelerometer and gyroscope	Yes	Wrist
Cheong *et al*^55^	Activity tracker	Android or iOS	Patron wristband	Urban S	Step count, walking distance, heart rate	Wristband accelerometer and heart rate sensor	No	Wrist
Patterson *et al*^56^	Activity tracker	Fitbit OS, Android or iOS	Fitbit wristband	Fitbit Flex	Step count, floors climbed, calories expended and active minutes	Wristband accelerometer	No	Wrist
Christiansen *et al*^58^	Activity tracker	Fitbit OS, Android or iOS	Fitbit wristband	Fitbit Zip	Step count	Wristband accelerometer	No	Waist belt
Paul *et al*^53^	App on smartphone	Android	Starfish App on Android smartphone	Samsung Galaxy S3	Step count	Smartphone accelerometer	No	Waist belt or pocket
Fusari *et al*^59^	App on smartphone and smartwatch	iOS and Apple Watch OS	OnTrack App on iPhone and Apple watch	iPhone and Apple watch	Upper limb movement	Smartwatch accelerometer	Yes	Wrist
Katzan *et al*^57^	Activity tracker	Fitbit OS, Android or iOS	Fitbit wristband	Fitbit Charge HR	Step count	Wristband accelerometer	No	Wrist
Klassen *et al*^48^	Activity tracker	Fitbit OS, Android or iOS	Fitbit wristband	Fitbit One	Step count	Wristband accelerometer	No	Ankle
Mackenzie *et al*^54^	Activity tracker	Fitbit OS, Android or iOS	Fitbit wristband	Fitbit Fire II	Overall activity (combination factoring quality of movement, frequency, duration, intensity and pattern)	Wristband accelerometer	No	Wrist
Toogood *et al*^49^	Activity tracker	Fitbit OS, Android or iOS	Fitbit wristband	Not specified	Step count	Wristband accelerometer	No	Ankle

RCTrandomised controlled trial

### Monitoring versus intervention

Wearable devices were used to monitor recovery and predict outcomes in six studies.^48^,^5054 56 57^ On the other hand, 12 studies used wearable technology as part of an intervention, aiming to improve rehabilitation outcomes.^42^,^4751^ Of these, the wearable devices provided real-time patient feedback in 10 studies,^42^,^4652 53 55 58 59^ and real-time physiotherapist monitoring in 6 studies.^42 46 47 51 55 59^ A summary of all study interventions is presented in table 3.

**Table 3:** Aims, outcomes and limitations of included studies

Study	Aim of study	Tech used for intervention or monitoring?	Follow-up	Primary outcomes	Results and conclusions	Limitations
RCTs						
Lawrie *et al*^44^	To assess the feasibility of delivering tailored activity feedback to patients who had a stroke using smartwatch activity monitors and assess retention rate, adherence and potential efficacy to inform potential RCT	Intervention	3 months	Feasibility, patient engagement and potential efficacy of wearable device PA intervention	11% recruitment and 48% retention rate at 3 months postdischarge. 80% adherence rate to wearing smartwatch. Baseline activity exceeded for 65% of days in feedback group compared with 55% in no-feedback group. Delivery of smartwatch RCT is feasible, however frequent support and guidance of ward staff are required to endure completeness of data and protocol adherence.	Study recruitment took place in China while study protocol and data analysis conducted in the UK, posing significant communication hurdles, with challenges to healthcare staff understanding and adhering to study protocol. Study was underpowered and authors were unable to perform a statistical analysis on the potential efficacy of the intervention.
Van der Walt *et al*^43^	Determine if step count feedback from a commercial activity tracker improves PA following THA or TKA	Intervention	6 months	Efficacy of feedback from activity tracker on the promotion of PA	Patients receiving step count feedback had significantly higher mean daily step counts (43% higher in week 1, 33% higher in week 2, 21% higher in week 6 and 17% higher at 6 months). Step count feedback from commercial activity tracker significantly increased activity levels post THA and TKA up to 6 months postoperatively.	30 patients were excluded from the study due to late arrival or delivery of their devices. Authors had concerns as to the accuracy of the activity monitor at low speeds, making results from the initial postoperative days potentially erroneous.
Crawford *et al*^42^	Determine the non-inferiority of a smartphone-based remote rehabilitation programme post TKA and PKA compared with traditional physiotherapy rehabilitation	Intervention	90 days	Efficacy and functional recovery outcomes of smart device based self-directed rehabilitation	No significant differences at 90 days in mean flexion, single leg stance, treatment, Time up and Go time, or need for manipulation under anaesthesia between the intervention and control groups. Control group had significantly higher physiotherapist input and more emergency department visits. The smartwatch/smartphone platform was non-inferior of clinically significant outcomes to traditional care models, while requiring less post-operative physiotherapy input. The platform has the potential of decreasing post-operative costs.	Heterogeneity in the standard of care across multiple centres involved in patient recruitment. Surgery and post-operative care protocols were not standardised across sites. No standardisation of prosthesis. Patients excluded if no access to compatible smartphone.
Tripuraneni *et al*^46^	Evaluate the impact of a self-directed rehabilitation programme post TKA on postoperative outcomes	Intervention	12 months	Efficacy and functional recovery outcomes of smart device based self-directed rehabilitation	No clinically significant difference was observed in PROMs, ROM or need for manipulation under anaesthesia between the control and intervention groups. Smart device driven self-directed physiotherapy achieved similar clinically relevant outcomes to formal physiotherapy, and surgeons can consider this as an appropriate alternative after TKA.	Patients excluded if no access to compatible smartphone. No economic evaluation was conducted for this wearable device intervention.
Kanai *et al*^47^	To evaluate the effect of accelerometer-based feedback on PA in hospitalised patients with ischaemic stroke	Intervention	Minimum 3 days until discharge	Efficacy of feedback from activity tracker on the promotion of PA	No significant difference in PA values between intervention and control groups at baseline measurement. Follow-up PA value in the intervention group was significantly higher than the control group. Exercise training combined with accelerometer-based feedback increased PA in hospitalised patients with ischaemic stroke.	Small sample size, and single centre study. Only patients who could walk without assistance were included, hence results not generalisable to wider stroke population. Follow-up period was not standardised and was different for all patients—determined by discharge from hospital date. Hence results reflect a short follow-up (inpatient stay) and long-term outcomes are not clear.
Wolk *et al*^45^	To monitor and increase postoperative mobilisation of patients after major visceral abdominal surgery, by providing continuous step count feedback using activity trackers	Intervention	5 days	Efficacy of feedback from activity tracker on the promotion of PA	Average step count during postoperative days 1–5 was significantly increased by feedback compared with the control group, as was the activity time. This effect was only observed for laparoscopic operations and not open surgery. Patients who achieved more than the median step count, had a significantly shorter hospital stay. Study demonstrates activity tracking and feedback can enhance perioperative mobilisation after laparoscopic surgery.	Very short follow-up duration—long-term effects of intervention uncertain. Significant heterogeneity in the operations included in the study (hepatopancreatic, gastrointestinal and colorectal) with likely varying impacts on patient mobility.
Non-randomised quasi-experimental and observational studies						
Lyman *et al*^50^	Assess feasibility of mobile devices to collect daily step count and PROMs to monitor recovery after THA and TKA	Monitoring	6 months	Feasibility and patient engagement with wearable device monitoring	78% PROMs completed, step data available for 92% of days for male and 86% of days for female patients. Adherence and completion rates satisfactory, supporting use of mobile devices for monitoring recovery post TJA.	Could not assess if patients were carrying devices all the time or where they were carried (eg, female patients carrying in a purse). Patients excluded if no access to compatible smartphone. Participants’ smartphones used—heterogeneous devices.
Broderick *et al*^52^	Assess feasibility and efficacy of remote PA intervention using wearable technology	Intervention	12 weeks	Feasibility and patient engagement with wearable device intervention	87% patients completed 12 weeks and 69% completed 24 weeks of intervention. Intervention increased functional capacity, improved QoL and PROMs. Remote PA intervention feasible and well received by patients with cancer.	Heterogeneous patient cohort (multiple different cancer types), small sample size and single-centre study. Patients excluded if no access to compatible smartphone.
Chae *et al*^51^	Develop and evaluate the efficacy of an HBR system using a smartwatch and smartphone	Intervention	18 weeks	Feasibility and efficacy of HBR with wearable devices	78% completion rate at 12 weeks and 55% at 18 weeks (higher than control group). Patients in the HBR group had improvement in the Wolf Motor Function Test, ROM of shoulder flexion and internal rotation. HBR using commercial wearables can improve upper limb function in stroke survivors.	Underpowered study with limited patient numbers. Patients excluded if no access to compatible smartphone. Discrepancy in number of participants in control and HBR groups (only 6 patients in control group included in data analysis).
Cheong *et al*^55^	Assess the feasibility and efficacy of remote tailored rehabilitation using smartphones and wearable devices	Intervention	12 weeks	Feasibility and efficacy of remote rehabilitation with wearable devices	74% intervention completion rate at 12 weeks. Improved lower extremity strength and cardiorespiratory endurance, and decreased fatigue, nausea and vomiting. A tailored rehabilitation programme using wearable devices improved physical capacity and treatment-related symptoms.	No control group included for comparison – improvement in treatment-related symptoms maybe a result of progression of treatment. Influence of different chemotherapy regimens was not considered.
Patterson *et al*^56^	Assess if consumer wearable devices can stratify patients by activity changes post TJA, to identify those needing targeted therapy	Monitoring	12 weeks	Monitoring recovery and prediction of postoperative outcomes	Wearable devices stratified patients into minimally changed and decreased activity post TJA. Decreased activity group had greater pain reduction postoperatively. Remote monitoring of wearable device data may facilitate real-time detection of early post-TJA problems.	Underpowered sample size and short follow-up period. Patients excluded if no access to compatible smartphone.
Christiansen *et al*^58^	Assess the feasibility, fidelity, safety and efficacy of PA intervention using activity sensor post-TKR	Intervention	6 months	Feasibility, fidelity, safety and efficacy of remote rehabilitation with wearable devices	84% retention rate in the intervention group. Adherence to intervention ranged from 45% to 60%. Intervention group patients had 1798 more steps/day, with 73.4 more minutes per week of moderate to vigorous PA at 6 months compared with the control group. A PA intervention using activity sensors is feasible, safe, demonstrates treatment fidelity and may increase PA.	Small sample size. Only patients who were interested in increasing the PA post-TKA were included, limiting the generalisability of the study sample.
Paul *et al*^53^	To assess the effectiveness of Starfish in patients who had a stroke—an app-based PA intervention using visual feedback, self-monitoring and social support	Intervention	6 weeks	Efficacy of wearable app-based intervention in improving PA	39% increase of average daily step count in the intervention group, compared with 20% decrease in the control group. Use of Starfish has potential to improve PA and health outcomes in stroke survivors.	Underpowered study with small sample size. Participants were young for a cohort of stroke survivors (mean age 56.3 years in the intervention group), limiting generalisability of findings. Assessor was not blinded to the group allocation. Short intervention duration.
Fusari *et al*^59^	Assess feasibility and acceptability of OnTrack in patients who had a stroke—an app-based wearable device intervention supporting upper limb rehabilitation	Intervention	12 weeks	Feasibility and acceptability of remote rehabilitation with wearable devices	50% intervention completion rate despite COVID-19 disruption. Intervention acceptable with compliance rate of 86%. OnTrack intervention was feasible and acceptable, demonstrating that a definitive RCT would be viable.	Underpowered study with small sample size and short intervention period. Significant disruption to study protocol due to COVID-19. Study did not assess clinical effectiveness of wearable system for patients who had a stroke.
Katzan *et al*^57^	Determine adherence of patients who had a stroke with wearing a consumer-grade activity tracker, assess its accuracy and evaluate patient experience	Monitoring	90 days	Feasibility and patient engagement with wearable device monitoring	84% mean daily adherence with activity monitor. Mean step count difference between device and manual tally was −1.8%. The use of consumer-grade activity tracker in patients with mild stroke is feasible with reasonable adherence and accuracy.	The study did not assess clinical effectiveness of the device for patients with stroke. Only patients with mild stroke included—not generalisable to more severe stroke. Underpowered study with small sample size.
Klassen *et al*^48^	To assess the accuracy of consumer-based physical activity monitor during rehabilitation of patients who had a stroke	Monitoring	4 weeks	Accuracy of consumer-grade wearable device in PA monitoring	Strong correlation between number of steps captured by the Fitbit One and StepWatch Activity monitors (research device with proven accuracy). Study provides preliminary evidence that Fitbit One can accurately measure walking steps early after stroke.	Underpowered study with small sample size, and short intervention period. Accuracy of StepWatch Monitor quoted at 96.1%. A truer gold standard would be a manual step count tally or video analysis of steps.
Mackenzie *et al*^54^	To evaluate the potential of commercially available activity trackers to assess and monitor post-treatment outcomes of frozen shoulder	Monitoring	14 days	Feasibility of wearable device assessment and post-treatment recovery monitoring	Mean activity counts using the Fitbit Fire II in the frozen shoulder limb significantly lowered pretreatment compared with the normal limb. Post-treatment, the wearable device detected significant improvement of activity in the previously frozen limb. Wrist-mounted activity trackers are sufficiently sensitive to detect difference in limb activity in patients affected by frozen shoulder, offering insights into disease burden and recovery.	Underpowered study with small sample size and short intervention period. Lack of correlation between activity measurements and PROMs during the study likely represents a type II error resulting from small sample size.
Toogood *et al*^49^	To assess the feasibility of remote mobility monitoring using activity trackers post-THA and define mobility recovery patterns	Monitoring	30 days	Feasibility of remote monitoring of mobility using activity trackers	89% compliance over a 30-day intervention period. Mean steps increased from 235 to 2563 over 30 days. Age <70 years, anterior surgical approach and discharge to home rather than to a nursing facility was associated with higher activity. Remote mobility monitoring post-THA using commercially available wearables is feasible and may allow early identification and intervention in slowly recovering patients	Small sample size, with futile subgroup analysis. Patient perceptions or acceptability of the technology was not evaluated. The accuracy of the Fitbit device was not assessed during this study.

HBRhome-based rehabilitationPAphysical activityPKApartial knee arthroplastyPROMspatient-reported outcome measuresQoLquality of lifeRCTrandomised controlled trialROMrange of motionTHAtotal hip arthroplastyTJAtotal joint arthroplastyTKAtotal knee arthroplasty

### Wearables in orthopaedics: improvement in patient outcomes?

#### Findings of randomised controlled studies in orthopaedics

Van der Walt *et al*^43^ conducted an RCT on patients undergoing total hip arthroplasty (THA) and total knee arthroplasty (TKA), evaluating the use of a Garmin Vivofit 2 activity tracker. Patients were randomised into two groups, with the intervention group participants able to view their daily step count and receive a daily step goal. The authors found that patients in the intervention group achieved higher activity levels after THA and TKA over 6 weeks (mean daily step count, 21% higher, p<0.03) and 6 months (17% higher, p<0.03), compared with patients not receiving feedback. A potential limitation of this study was that authors had concerns as to the accuracy of the activity monitor at low speeds, making results from the initial postoperative days potentially erroneous.

Crawford *et al*^42^ and Tripuraneni *et al*^46^ both performed RCTs on patients undergoing knee arthroplasty, evaluating the use of the ‘MyMobility’ app on iPhones and Apple watches. The app measures step count, flights of stairs climbed, stand hours and heart rate, as well as encourages patients to complete self-directed exercise modules. Crawford *et al*^42^ demonstrated non-inferiority of the wearable platform for clinically significant outcomes compared with usual postoperative care, while decreasing postoperative physiotherapy and emergency department visits. One significant limitation of this study was that surgery and postoperative care protocols were not standardised across sites. Similarly, Tripuraneni *et al*^46^ also demonstrated non-inferiority with the wearable platform, with postoperative outcomes including manipulation under anaesthesia, range of knee movement and patient-reported outcome measures (PROMs) not being different to traditional physiotherapy. This demonstrates how wearables can potentially substitute traditional physiotherapy and decrease the strain on the health service.

#### Findings of observational studies in orthopaedics

Christiansen *et al*^58^ undertook a study on patients undergoing unilateral TKR. Aside from standard physiotherapy, patients in the intervention arm were given a Fitbit device to monitor step count, with monthly phone calls. Despite a small sample size, the authors demonstrated that patients in the intervention group accumulated more daily steps and spent 73 more minutes a week engaging in moderate-to-vigorous physical activity at 6 months.

Lyman *et al*^50^ evaluated the ‘Moves App’ for monitoring postoperative THA and TKA recovery. The app uses an iOS or Android smartphone’s accelerometer to track patient step count, with a feature allowing patients to complete PROMS. The authors demonstrated that tracking patients’ mobility and monitoring their rehabilitation was feasible. A limitation was that authors were not able to determine if participants were carrying devices all the time, or how they were carrying them. Patterson *et al*^56^ used a Fitbit tracker to stratify patients by change in activity pre and post THR and TKR surgery. By monitoring step count, the authors identified two clusters of patients that predicted their likelihood of experiencing improvements in pain. This would allow real-time screening of early problems following THR and TKR. Both Mackenzie *et al*^54^ and Toogood *et al*^49^ evaluated the Fitbit in an orthopaedic cohort. Mackenzie *et al*^54^ found the Fitbit allowed clinicians to assess effectiveness and monitor recovery of patients with frozen shoulder post surgical and non-surgical interventions. The authors noted a lack of correlation between activity measurements and PROMs during the study, likely representing a type II error resulting from small sample size. Toogood *et al,*^49^ on the other hand, demonstrated that the Fitbit allowed clinicians to evaluate functional status and recovery objectively using daily step counts between follow-up visits.

### Do wearables improve rehabilitation outcomes following stroke?

#### Findings of RCTs in stroke medicine

Lawrie *et al*^44^ conducted an RCT on stroke survivors, and participants were provided Android smartwatches which estimate physical activity using an accelerometer. Participants were divided into real-time activity feedback and a no-feedback groups. The authors found that patients receiving goal-directed feedback exceeded their baseline activity in 65% of days compared with 55% in the non-feedback group. A significant limitation of this study was that patients were recruited in China, while study conception and data analysis was conducted in the UK, with communication barriers affecting adequate intervention delivery.

Kanai *et al*^47^ undertook an RCT to assess the effect of using a Fitbit device on improving physical activity among stroke survivors. Patients in the intervention group received step count feedback in conjunction with a supervised rehabilitation programme, whereas the control group received no feedback. The results demonstrated a significant improvement in the average number of steps (5180.5 steps/day vs 3113.6 steps/day in the intervention group vs control group, respectively, p=0.0003). A limitation was that only patients able to mobilise independently were included, with the results not being generalisable to the wider stroke population.

#### Findings of observational studies in stroke medicine

Chae *et al*^51^ developed a home-based rehabilitation system for stroke survivors using an LG Smartwatch. Patients in the intervention arm had a significant improvement in their Wolf Motor Function Score (p=0.02), and improved shoulder flexion (p=0.004) and internal rotation (p=0.03). Aside from being underpowered, one limitation of this study was a discrepancy in the group sizes, with only six patients in the control group being included in the data analysis. Paul *et al*^53^ tested an interactive app called ‘STARFISH’ on stroke survivors. The app gamifies step count, converting it into a fish that swims and blows bubbles when patients move. Starfish also facilitates social support, allowing patients to see each other’s fish, to increase motivation. The authors noted a 39.3% increase in step count within the intervention group compared with a 20.2% decrease within the control group. With a mean participant age of 56.3 years, a limitation of this study is that the younger participant cohort does not reflect a typical cohort of patients who had a stroke.

Fusari *et al*^59^ evaluated the use of an Apple Watch and iPhone app called ‘OnTrack’ among stroke survivors. The app encourages patients to increase their arm movement, with real-time remote physiotherapist monitoring and motivational messaging. Although this was a feasibility study that did not assess OnTrack’s influence on patient outcomes, the authors demonstrated that the intervention was both feasible and acceptable by participants.

Katzan *et al*^57^ evaluated the feasibility and adherence of Fitbit devices for monitoring mobility in stroke survivors. While underpowered, the study demonstrated that a consumer-grade activity monitor was feasible with an 83.6% adherence over the 90-day period. Klassen *et al*^48^ evaluated the accuracy of Fitbit One devices in monitoring walking activity of stroke inpatients. The authors found that the device accurately measured walking steps during early rehabilitation.

### Do wearables improve mobilisation post major abdominal surgery?

Only one study assessed the use of wearables in General Surgery. This was an RCT conducted by Wolk *et al*,^45^ assessing the effect of step count feedback via a Polar Loop activity tracker on postoperative mobilisation in patients undergoing major abdominal visceral surgery. The study demonstrated a significant increase in mobility (cumulative step count 9867 vs 6103, p=0.037) and activity time (483 min vs 348 min, p=0.037) during the first five postoperative days in patients receiving feedback. This effect was only observed for patients undergoing laparoscopic surgery, and not open abdominal surgery. The main limitation was the short follow-up of 5 days, with uncertainty regarding long-term effects of the intervention.

### Can wearables improve rehabilitation in patients with cancer?

Broderick *et al*^52^ undertook a single-arm longitudinal feasibility study using a Fitbit device in conjunction with physiotherapist input to set physical activity goals for cancer survivors. While this was a heterogenous group of patients, the authors did find an improvement in patients’ functional capacity (6 min walk test, mean 557 m at baseline vs 597 m at 24 weeks, p=0.002) and quality of life (physical functioning measure, score 85 at baseline vs 90 at 24 weeks, p=0.0001) following the intervention. Cheong *et al*^55^ used an Urban S Patron wristband, to provide step count feedback, walking distance and heart rate, in conjunction with an individualised rehabilitation exercise programme for chemotherapy patients. After 12 weeks, the authors noted a significant improvement in both lower extremity strength (chair-to-stand test, mean 17.3 stands at baseline vs 20.9 at 12 weeks, p<0.001) and cardiorespiratory endurance (2 min walk test, mean 167 m at baseline vs 182 m at 12 weeks, p<0.001). Both studies investigating wearables in patients with cancer highlighted positive rehabilitation outcomes.

### Risk of bias in individual studies

The Cochrane Methods RoB 2 tool was used to assess the risk of bias in all six RCTs. There were some concerns of bias in at least one domain in all RCTs, with blinding of participants and the research team being a common reason for concern. None of the RCTs were found to have high risk of bias in any domain. The Cochrane Methods ROBINS-I tool was used to assess the risk of bias in the remaining 12 observational studies. Only one study by Lyman *et al*^50^ had serious risk of bias in the selection of participants domain. The remaining studies had low or moderate risk of bias across all seven domains. None of the non-randomised studies were found to have critical risk of bias in any domain (table 4).

**Table 4:** Summary of risk of bias assessment of included studies

Study	Tool used	Risk of bias judgement
Lyman *et al*^50^	ROBINS-I	Serious risk of bias in the selection of participants domain
Broderick *et al*^52^	ROBINS-I	Low or moderate risk of bias in all domains
Chae *et al*^51^	ROBINS-I	Low or moderate risk of bias in all domains
Cheong *et al*^55^	ROBINS-I	Low or moderate risk of bias in all domains
Patterson *et al*^56^	ROBINS-I	Low or moderate risk of bias in all domains
Christiansen *et al*^58^	ROBINS-I	Low or moderate risk of bias in all domains
Paul *et al*^53^	ROBINS-I	Low or moderate risk of bias in all domains
Fusari *et al*^59^	ROBINS-I	Low or moderate risk of bias in all domains
Katzan *et al*^57^	ROBINS-I	Low or moderate risk of bias in all domains
Klassen *et al*^48^	ROBINS-I	Low or moderate risk of bias in all domains
Mackenzie *et al*^54^	ROBINS-I	Low or moderate risk of bias in all domains
Toogood *et al*^49^	ROBINS-I	Low or moderate risk of bias in all domains
Lawrie *et al*^44^	RoB 2	Some concerns in the missing outcomes and selection of reported results domains
Van der Walt *et al*^43^	RoB 2	Some concerns in the deviation from the intended intervention domain
Crawford *et al*^42^	RoB 2	Some concerns in the deviation from the intended intervention domain
Tripuraneni *et al*^46^	RoB 2	Some concerns in the measurement of outcomes domain
Kanai *et al*^47^	RoB 2	Some concerns in the deviation from the intended intervention domain
Wolk *et al*^45^	RoB 2	Some concerns in the deviation from the intended intervention domain

RoB 2Methods Risk of Bias in Randomised Trials V.2ROBINS-IRisk of Bias in Non-Randomised Studies of Interventions

### GRADE evidence profile of included studies

Table 5 summarises the GRADE evidence profile of all included RCTs and non-randomised studies, categorised by medical discipline. The three Orthopaedics RCTs demonstrated the highest level of certainty that wearables were a potential alternative to conventional physiotherapy, with two RCTs^42 46^ highlighting non-inferiority of wearables in postoperative rehabilitation.

**Table 5:** Summary of GRADE evidence profile for the included RCTs and non-randomised studies

Number of studies	Combined number of patients	Main findings	GRADE evidence profile					
			Risk of bias	Imprecision	Inconsistency	Indirectness	Other considerations	Overall certainty
**Orthopaedics RCTs**								
3	952	Non-inferiority of wearables’ self-directed rehabilitation compared with traditional physiotherapy Wearables are potential alternatives to traditional physiotherapy Wearable-driven feedback increases physical activity	Low	Low	Low	Low	None	High
**Stroke medicine RCTs**								
2	72	Wearables-driven feedback increases physical activity	Moderate	High	Low	High	None	Low
**General surgery RCTs**								
1	132	Wearable-driven feedback increases physical activity post open abdominal surgery	Low	Moderate	Low	Low	None	Moderate
**Oncology non-randomised quasi-experimental and observational studies**								
2	120	Wearables were acceptable to patients with cancer with high intervention completion rates Remote self-directed rehabilitation using wearables is feasible	Moderate	Low	Low	Moderate	None	Very Low
**Orthopaedics non-randomised quasi-experimental and observational studies**								
5	384	Wearable-driven feedback increases physical activity Wearables can monitor patient postoperative progress remotely Remote wearable-driven physical therapy is feasible in postoperative patients	High	Low	Low	Low	None	Very Low
**Stroke medicine non-randomised quasi-experimental and observational studies**								
5	94	Wearable-driven feedback increases physical activity post stroke Remote wearable-driven physical therapy is feasible and acceptable to patients who had a stroke	Moderate	Low	Low	Moderate	None	Very Low

GRADEGrading of Recommendations, Assessment, Development and EvaluationRCTrandomised controlled trial

The two stroke RCTs were rated down due to the higher risk of bias across two Cochrane Methods Risk of Bias in Randomised Trials V.2 domains as well as the imprecision with missing outcome reporting in Lawrie *et al*’s RCT.^44^ The second RCT by Kanai *et al*^47^ contributed to the downrating with the indirectness of the study methodology, only including patients who were able to walk independently, something which is not generalisable to the wider stroke population. Imprecision, with only one General Surgery RCT led to the evidence certainty being rated down to moderate for the use of wearables in the postoperative general surgery setting.

Among the non-randomised studies, all evidence certainty profiles were downrated to very low across oncology, orthopaedics and stroke studies. This was a combination of moderate-to-high risk of bias, as well as indirectness of the study populations in the oncology and stroke studies.

## Discussion

The advantages of mHealth and the use of wearables in healthcare are well documented in the literature.^22^,^25^ With a recent shift towards the utilisation of consumer-grade wearables in measuring physical activity in healthcare,^38^ this study is one of the first to review the multidisciplinary use of consumer-grade off-the-shelf devices in physiotherapy and rehabilitation, which has widespread applicability.

The aim of this review was to evaluate whether consumer-grade wearables positively impact rehabilitation outcomes, and to categorise all wearables being used, including the disciplines and medical conditions for which they are being used. All RCTs and non-randomised studies demonstrated improved rehabilitation outcomes and intervention feasibility,^42^,^4751^ with two RCTs demonstrating non-inferiority of wearables when compared with traditional physiotherapy.^42 46^ This has significant clinical impact when resources are limited, for example, due to lack of physiotherapists, where wearables have the potential to drive self-directed rehabilitation. Among the observational studies, there was similarly an agreed consensus that wearables have potential to improve clinicians’ objective understanding and monitoring of patient recovery and rehabilitation, as well as screening of patients at risk who may require more targeted input.^48^,^5054 56 57^

The current review demonstrates discipline-wide potential advantages of mHealth and the use of wearables in patient rehabilitation, especially when used in conjunction with traditional physiotherapy. Having said that, 12 of the included studies were non-RCTs, which affected the overall certainty of evidence as per the GRADE process. Only the Orthopaedics RCTs were deemed to have high certainty evidence, with the remaining RCTs being rated down due to risk of bias, imprecision and indirectness. The evidence for the non-randomised studies was rated as low or very low highlighting that better designed studies and more RCTs, with greater participant numbers are required, particularly in the fields of Stroke, Oncology and General Surgery. Moreover, detailed economic evaluations are required to draw firm conclusions prior to the widespread recommendation of wearables and self-directed rehabilitation. A unified cross-discipline consensus on the use of wearables in physical rehabilitation is needed to streamline services and allow for seamless integration. Furthermore, a review is required on the barriers and facilitators to the adoption of wearables, based on patient and medical professional feedback.

Orthopaedics was most widely researched, closely followed by stroke medicine. This may be a reflection of physiotherapy being heavily relied on within these specialities.^60^ Moreover, limited resources may be driving clinicians to turn to wearables as potential solutions. The most widely studied device was the Fitbit, evaluated in eight studies.^47^,^4952 54 56^ This was followed by Apple’s iOS and Watch-OS in four studies.^42 46 50 59^ Google’s Android platform was used in three studies.^44 50 53^ Less well-studied devices included the Polar Loop Activity Tracker, the Garmin Vivofit 2 and the Patron Urban S wristband.^43 45 55^

Of note, most studies used standard activity outcome data from wearables including step count, flights climbed and heart rate (table 2), whereas only three studies^44 51 59^ focused on more specific derived outcomes, such as limb movement or estimated overall body movement. This highlights a potential need to develop condition or discipline-specific measures, possibly delivered by machine learning to classify specific activity and movement beyond basic outcome measures.

Seven studies^4246 50^,^52 55 56^ including two RCTs^42 46^ excluded patients who did not have access to smartphones compatible with study protocols. While increasing numbers of the population access smartphones,^19^ excluding patients that do not could bias the results and skew intervention compliance in favour of patients already familiar with smartphones. Moreover, if owning a smart device becomes mandatory to access these benefits, this would pose ethical dilemmas with lower socioeconomic status patients being disadvantaged. A study by Kim *et al*^61^ investigating the use of wearables among children, noted that black children and those from lower socioeconomic status were less likely to participate and wore devices for significantly less time. This further highlights the importance of considering socioeconomic status and the social determinants of health, not only when designing research studies, but also when implementing technology in real patient cohorts.

A common trend among the stroke studies was the exclusion of patients with severe strokes. Though the results of these studies may not be generalisable to a real-world stroke cohort, this may highlight a potential role for a tiered approach in adopting technology in physiotherapy, with wearables and self-directed rehabilitation being used for mild-to-moderate disability, freeing up face-to-face hospital physiotherapy for patients with more severe disability.

We recognise that there are limitations to this review. The studies included are heterogeneous, with several multidisciplinary specialities, a variety of different treated conditions, using different wearables and various interventions. This undoubtedly weakens conclusions on specific benefits of each device. However, though each device varies in brand, the underlying technology is similar, using an accelerometer or a gyroscope. Moreover, a real-world patient cohort is similarly heterogeneous, and if we strive to find solutions to limited resources, using consumer-grade wearables is a pragmatic and potentially cost-effective approach. A further limitation lies in the small sample size within the included studies. Though three of the studies tested wearable technologies on more than 250 patients,^42 46 50^ eight of the studies were relatively underpowered and evaluated wearables on less than 25 patients.^44 48 51 53 54 56 57 59^ Additionally, the maximum follow-up duration was 12 months in only one study,^46^ hence conclusions cannot be drawn on the long-term impact of consumer-grade wearables. Finally, there are limitations within the level of evidence included in this review. Twelve studies were level III or IV quasi-experimental or observational studies, and six were level II RCTs. All six RCTs had some concern for bias in at least one domain, and of the non-randomised experimental and observational studies, one had serious risk of bias in the selection of participants domain. This is reflected in the GRADE evidence certainty for the included studies (table 5).

The studies in this review demonstrate that consumer-grade wearables have the potential to be used as adjuncts to traditional physiotherapy, and potentially as alternatives for self-directed rehabilitation. Having said that, better designed studies, and larger RCTs appropriately informed by sample size calculations, with a focus on economic evaluations of these technologies are needed before a case can be made for their widespread adoption in healthcare settings. This is an emerging field, and likely to influence the future of self-directed rehabilitation.

## supplementary material

10.1136/bmjopen-2024-084086 online supplemental file 1

## Data Availability

All data relevant to the study are included in the article or uploaded as supplementary information.
