# Supplementary material for: Use of commercially available wearable devices for physical rehabilitation in healthcare: a systematic review
Source: BMJ Open. 2024 Nov 7;14(11):e084086. doi: 10.1136/bmjopen-2024-084086 (PMC11552580; doi:10.1136/bmjopen-2024-084086)
Supplement: online supplemental file 1 [file bmjopen-14-11-s001.pdf]

THE USE OF COMMERCIALLY AVAILABLE WEARABLE DEVICES FOR PHYSICAL REHABILITATION IN HEALTHCARE: A SYSTEMATIC REVIEW

Appendix A. Search Strategies

Embase and MEDLINE

(“wearable technology” [MeSH] OR “wearable sensor” [MeSH] OR “wearable device” [MeSH] OR “wearable monitor” [MeSH] OR “wearable tracker” [MeSH] OR “activity monitor” [MeSH] OR “activity sensor” [MeSH] OR “activity tracker” [MeSH])

AND

(“physiotherapy” [MeSH] OR “physical therapy” [MeSH] OR “rehabilitation” [MeSH] OR “rehab” [MeSH] OR “exercise programme” [MeSH] OR “exercise program” [MeSH] OR “physical activity” [MeSH])

Cochrane Library

(“wearable technology” OR “wearable sensor” OR “wearable device” OR “wearable monitor” OR “wearable tracker” OR “activity monitor” OR “activity sensor” OR “activity tracker”)

AND

(“physiotherapy” OR “physical therapy” OR “rehabilitation” OR “rehab” OR “exercise programme” OR “exercise program” OR “physical activity”)

Web of Science

(ALL=(wearable technology) OR ALL=(wearable sensor) OR ALL=(wearable device) OR ALL=(wearable monitor) OR ALL=(wearable tracker) OR ALL=(activity monitor) OR ALL=(activity sensor) OR ALL=(activity tracker))

AND

(ALL=(physiotherapy) OR ALL=(physical therapy) OR ALL=(rehabilitation) OR ALL=(rehab) OR ALL=(exercise programme) OR ALL=(exercise program) OR ALL=(physical activity))
