# Supplementary material for: Use of commercially available wearable devices for physical rehabilitation in healthcare: a systematic review
Source: BMJ Open. 2024 Nov 7;14(11):e084086. doi: 10.1136/bmjopen-2024-084086 (PMC11552580; doi:10.1136/bmjopen-2024-084086)
Supplement: Supplementary file 2 [file bmjopen-2024-084086.reviewer_comments.pdf]

PEER REVIEW HISTORY

BMJ Open publishes all reviews undertaken for accepted manuscripts. Reviewers are asked to complete a checklist review form and are provided with free text boxes to elaborate on their assessment. These free text comments are reproduced below.

ARTICLE DETAILS

Title (Provisional)

The Use of Commercially Available Wearable Devices for Physical Rehabilitation in Healthcare: A Systematic Review

Authors

Latif, Ahmed; Al Janabi, Hasaneen Fathy; Joshi, Meera; Fusari, Gianpaolo; Shepherd, Leila; Darzi, Ara; Leff, Daniel

---

VERSION 1 - REVIEW

Reviewer 1

Name Zadro, Joshua

Affiliation University of Sydney, Institute for Musculoskeletal Health, School of Public Health

Date 19-Jan-2024

COI No competing interests

I thank the authors for the opportunity to review this interesting paper investigating the effects of wearable technology on patient rehabilitation outcomes. The manuscript is very well-written and organised, and mostly well-reported. Below are a few comments that could improve this paper.

Abstract

- It would be more beneficial for the reader if the findings of RCTs were reported separately to quasi-experimental studies and observational studies (non-RCTs). This will help readers understand where the gaps are for RCTs and to better understand the certainty of evidence.

- Certainty of evidence (using GRADE) should also be incorporated into the conclusion of the review, strengths and weakness of the study, and throughout the manuscript when findings are reported (e.g. results, discussion, conclusion)

Introduction

- The population needs to be mentioned in the aims

Methods

- Please include the full search strategy as an appendix for replication and updating purposes

- Please include a GRADE assessment in the methods and frame the results, discussion, and conclusions in light of the certainty of evidence

- More information about the analysis is needed in the methods. Was a meta-analysis possible for some outcomes?

Results

- Please separate the results by findings of RCTs vs. non RCTs. Mixing them together makes it difficult to understand the certainty of evidence of the findings. If there were no RCTs for a particular population or outcome, please make this clear. Whether a study is an RCT or non-RCT also needs to be clear in the tables that explain the findings (not just the first table). This will save the reader from flipping back and forth between tables to understand the study design for particular findings

- Similarly in the discussion/conclusion, the authors should focus on the findings of RCTs and make it clear when they are discussing findings from non-RCTs

---

**Reviewer**	**2**
**Name**	**Veerubhotla, Akhila**
**Affiliation**	**NYU Langone Health, Rehabilitation Medicine**
**Date**	**27-Feb-2024**
**COI**	**none**

This systematic review aims to summarize studies that used commercially available wearable technology for physiotherapy post acute disorders or surgery. This review is timely and needed given that wearable technology is gaining traction for continuous monitoring and personalized care provision. The study followed PRISMA guidelines and is registered in PROSPERO. Risk of bias is assessed for all selected studies. The methods are adequately described. The results are well presented and the tables are easy to read and understand. The discussion is to the point and includes major limitations of the review. The manuscript is very well written and structured.

**Reviewer**	**3**
**Name**	**Dobkin, Bruce**
**Affiliation**	**UCLA, Neuroscience**
**Date**	**29-Mar-2024**
**COI**	**none**

The investigators chose a heterogeneous group of acute diseases/surgeries and associated impairments to review a range of outcomes during subacute rehabilitation that deployed commercially available, wearable accelerometers to quantify activity. They do not state why they chose only to assess the possible value of sensors within their stated preconditions.

The search strategy for articles thus limited itself to a small subgroup of the efficacy trials of wearables that are available in the literature of randomized clinical trials (e.g., use of other sensors such as Opals, Actigraph, laboratory-designed accelerometers with additional components) for monitoring, feedback, telerehab, and outcome measures during the rehabilitation of acute and chronic impairments/disabilities. The investigators draw heavily on quasi-experimental and observational studies that do not seem to adequately back up their conclusions. What the presented studies do reveal is that wearable sensors are feasible and acceptable to wear, which has been shown many times.

Abstract:

The Conclusion makes what seems to be an overly positive statement, not backed by the research designs and other limitations of the studies presented. While economic considerations are worthy of addressing, the immediate emphasis for research needs to be on validity and efficacy of sensor data within disease/impairment groups and for particular rehabilitation interventions. The review, for example, barely touches upon the clinically important activity variables and amounts of change in activity that the wearables ought to demonstrate to supplement conventional outcome and monitoring measures. No conclusions were drawn about specific ways sensors might serve as an adjunct to traditional physiotherapy interventions and in what ways and under what circumstances sensors might serve as alternatives for self-directed rehabilitation.

Strengths and Limitations of this Study:

While this paper may be the first cross-discipline systematic review of commercially wearable devices for subacute rehabilitation in healthcare, the only certainty is that the studies used primarily IMUs with accelerometers and data collection methods about movement in mostly underpowered pilot studies in a variety of ways for a variety of purposes. This may be interesting, but primarily suggests that the applications need better designed trials before coming into wider use. That the report "Demonstrates a clear advantage for the use of wearables in rehabilitation" is debatable given all the <50-subject, non-randomized, non-blinded, flawed in design or execution, etc., studies presented. No one would consider this collection of studies as worthy of a meta-analysis of possible efficacy.

Methods:

I wonder whether additional search terms, including IMUs or accelerometers AND activity or walking, might have broadened the studies found.

Discussion:

Please include more information about the use of tracking devices on the wrist versus the ankle versus a pocketed smartphone for step counts and distance walked. One investigator in one of the papers said inaccuracy at slow speeds was likely and one study use an ankle device. There is considerable evidence that only ankle sensors are reliable in detecting stepping at <0.5 m/s and that an algorithm must be created to enable recognition of slow walking.

Reliability of step counts seems to have been the only aim of the Klassen and the Katzen Fitbit studies. Indeed, it is unclear why these two studies were chosen for inclusion in your review, since, while the Fitbit accurately measured steps taken during rehabilitation of stroke, no assessment was made relevant to the value of the device for activity monitoring of rehabilitation or outcomes.

Discuss differences between statistically significant changes, e.g., in step counts or distance walked or upper extremity movements and clinically meaningful changes within the reviewed studies.

Did any of the studies assess walking speed based on the devices used? If so, what might be the value and limitations of this measure?

The STARFISH study is an example of uncertainty about which intervention was most valuable or whether all the interventions that included step monitoring may be necessary. Here, social support may have been as important as the bubbling fish for the experimental group. The same holds for the two quasi-experimental cancer studies – was feedback from sensors a critical component of improvements or was the rehabilitation and added support from the research group sufficient? These trials would not appear to add experimental support to your conclusions about the value of activity sensing.

In the Lawrie study, aside from other design problems, the difference of 10% more than baseline activity may not be clinically important. Any outcome for an RCT, of course, ought to be considered for statistical significance in its outcomes and whether differences represent a clinically important change. As in many other chosen papers, such issues limit your ability to draw conclusions about the value of activity sensing.

Regarding your methods to detect bias, please discuss the examples above that may contribute to actual bias in how you rated the studies. It is not always clear how the sensor data was related to any change in outcomes, especially given that most of the uncontrolled studies seem to have included multiple interventions – physiotherapy, inherent social support and personalized care, motivational incentives associated with participation in a study aimed at increasing activity, etc.

Conclusion:

You state: “The current review clearly demonstrates many discipline-wide potential advantages of mHealth and the use of wearables in patient rehabilitation, at the least when used in

conjunction with traditional physiotherapy, and at best as feasible cost-effective alternatives."

Although I too am a fan of mHealth and activity sensing for specific purposes, your presentation leaves this reader less than satisfied that the chosen studies and your methods of evaluation adequately support this conclusion. A bit later, you state that wearables require further study as feasible cost-effective alternatives.

In the final paragraph, you state that activity sensors can serve as an adjunct to traditional physiotherapy. What do you mean and state how, based on your chosen papers.

You also state that, at best, commercial wearables can be an alternative for self-directed rehabilitation. What do you mean and state how, based on your chosen papers.

Or is this conclusion meant to convey only an aspiration for acute rehabilitation wearables?

VERSION 1 - AUTHOR RESPONSE

Reviewer: 1
Mr. Joshua Zadro, University of Sydney
Comments to the Author:
I thank the authors for the opportunity to review this interesting paper investigating the effects of wearable technology on patient rehabilitation outcomes. The manuscript is very well-written and organised, and mostly well-reported. Below are a few comments that could improve this paper.

Authors' Response: We thank you for your review and for confirming that the paper is of interest, well-written and well-organised.

1. Abstract
- It would be more beneficial for the reader if the findings of RCTs were reported separately to quasi-experimental studies and observational studies (non-RCTs). This will help readers understand where the gaps are for RCTs and to better understand the certainty of evidence.
- Certainty of evidence (using GRADE) should also be incorporated into the conclusion of the review, strengths and weakness of the study, and throughout the manuscript when findings are reported (e.g. results, discussion, conclusion)

Authors' Response: We have updated the presentation of results for RCTs and Non-RCTs separately as per your recommendation. This is highlighted in the abstract, and updated in the revised results tables in which we subcategorise RCT vs Non-RCT, and in the discussion sections in which we discuss the results of these studies separately.

We have also added a certainty of evidence section, as advised, using GRADE. This is reflected in the abstract, methods, results (and new Table 5), as well as the discussion.

2. Introduction
The population needs to be mentioned in the aims

Authors' Response: The aims have been updated in the revised R1 manuscript to include the population (page 8).

3. Methods
Please include the full search strategy as an appendix for replication and updating purposes

Authors' Response: We have included a detailed search strategy as a supplementary file, Appendix A.

4. Please include a GRADE assessment in the methods and frame the results, discussion, and conclusions in light of the certainty of evidence

Authors' Response: We have amended the manuscript and utilised the GRADE assessment. This is now reflected throughout the manuscript, within the Abstract, Methods, Results section, Table 5 and Discussion.

5. More information about the analysis is needed in the methods. Was a meta-analysis possible for some outcomes?

Authors' Response: The included studies had significant population and study design heterogeneity, with each study testing a different wearable, a different condition and with a variety of outcome measures. A meta-analysis was not possible. We have updated this in the revised R1 manuscript to include why meta-analysis was not possible, on pages 11. We now state as follows: *"Given the heterogeneity of study populations, design, recorded outcomes, wearables tested and conditions treated, a meta-analysis was not possible"*.

6. Results
Please separate the results by findings of RCTs vs. non RCTs. Mixing them together makes it difficult to understand the certainty of evidence of the findings. If there were no RCTs for a particular population or outcome, please make this clear. Whether a study is an RCT or non-RCT also needs to be clear in the tables that explain the findings (not just the first table). This will save the reader from flipping back and forth between tables to understand the study design for particular findings
- Similarly in the discussion/conclusion, the authors should focus on the findings of RCTs and make it clear when they are discussing findings from non-RCTs

Authors' Response: We have updated the abstract, results, tables and discussion sections in the revised R1 manuscript to separate the findings of RCTs and non-RCTs. For example Table 2, "Characteristics of wearable technologies utilised in included studies" on page 16 is now divided under the headings of "Randomised Controlled Trials" and "Non-Randomised Quasi-Experimental and Observational Studies".

<u>Reviewer: 2</u>
Dr. Akhila Veerubhotla, NYU Langone Health
Comments to the Author:
This systematic review aims to summarize studies that used commercially available wearable technology for physiotherapy post acute disorders or surgery. This review is timely and needed given that wearable technology is gaining traction for continuous monitoring and personalized care provision. The study followed PRISMA guidelines and is registered in PROSPERO. Risk of bias is assessed for all selected studies. The methods are adequately described. The results are well presented and the tables are easy to read and understand. The discussion is to the point

and includes major limitations of the review. The manuscript is very well written and structured.

Authors' Response: Many thanks for your review and feedback especially on the importance and timeliness of this work.

Reviewer: 3
Dr. Bruce Dobkin, UCLA
Comments to the Author:

1. The investigators chose a heterogeneous group of acute diseases/surgeries and associated impairments to review a range of outcomes during subacute rehabilitation that deployed commercially available, wearable accelerometers to quantify activity. They do not state why they chose only to assess the possible value of sensors within their stated preconditions. The search strategy for articles thus limited itself to a small subgroup of the efficacy trials of wearables that are available in the literature of randomized clinical trials (e.g., use of other sensors such as Opals, Actigraph, laboratory-designed accelerometers with additional components) for monitoring, feedback, telerehab, and outcome measures during the rehabilitation of acute and chronic impairments/disabilities. The investigators draw heavily on quasi-experimental and observational studies that do not seem to adequately back up their conclusions. What the presented studies do reveal is that wearable sensors are feasible and acceptable to wear, which has been shown many times.

Authors' Response: Many thanks for your review and feedback, for which we are extremely grateful. Our review from the outset sought to explore only consumer grade wearables. These are tools that are already in the public sector and accessible to patients. With stretched physiotherapy services that often impact patient recovery, there is a pressing need for tools to aid current protocols, that are present and ready to use in the public domain, such as consumer grade wearables. Whilst we agree there is ample research using non-consumer grade wearables, such as laboratory based accelerometers, these have largely been confined to research settings. We have sought to make this clearer in the revised introduction section of the revised R1 manuscript (page 7). The use of consumer grade wearables such as FitBit and Apple Watches in rehabilitation is an emerging field of interest, given how accessible these sensors are, and hence there are limited studies with small participant numbers. There is of course a key role for laboratory based sensors in the development and advancement of this field, and how these can inform consumer grade wearables in the future. This is perhaps outside the remit of this current review and should be the subject of future reviews.

2. Abstract:
The Conclusion makes what seems to be an overly positive statement, not backed by the research designs and other limitations of the studies presented. While economic considerations are worthy of addressing, the immediate emphasis for research needs to be on validity and efficacy of sensor data within disease/impairment groups and for particular rehabilitation interventions. The review, for example, barely touches upon the clinically important activity variables and amounts of change in activity that the wearables ought to demonstrate to supplement conventional outcome and monitoring measures. No conclusions were drawn about specific ways sensors might serve as an adjunct to traditional physiotherapy interventions and in what ways and under what circumstances sensors might serve as alternatives for self-directed rehabilitation.

Strengths and Limitations of this Study:

While this paper may be the first cross-discipline systematic review of commercially wearable devices for subacute rehabilitation in healthcare, the only certainty is that the studies used primarily IMUs with accelerometers and data collection methods about movement in mostly underpowered pilot studies in a variety of ways for a variety of purposes. This may be interesting, but primarily suggests that the applications need better designed trials before coming into wider use. That the report "Demonstrates a clear advantage for the use of wearables in rehabilitation" is debatable given all the <50-subject, non-randomized, non-blinded, flawed in design or execution, etc., studies presented. No one would consider this collection of studies as worthy of a meta-analysis of possible efficacy.

Authors' Response: Many thanks for this feedback. We have acknowledged in the revised R1 manuscript that the quality of evidence of included studies is low on the whole (GRADE Evidence Profile, pages 28-29) and within the discussion, page 32, we state *"twelve of the included studies were non-RCTs, which affected the overall certainty of evidence as per the GRADE process. Only the orthopaedics RCTs were deemed high certainty evidence, with the remaining RCTs being rated down due to risk of bias, imprecision and indirectness. The evidence for the non-randomised studies was rated as low or very low highlighting that better designed studies and more RCTs, with greater participant numbers are required particularly in the fields of stroke, oncology and general surgery.".*

We have also revised and toned down the conclusion to be more balanced and acknowledge the need for better designed studies (pages 33-34), stating "*The studies in this review demonstrate that consumer-grade wearables have the potential to be utilised as adjuncts to traditional physiotherapy, and potentially as alternatives for self-directed rehabilitation. Having said that, better designed studies, and larger RCTs appropriately informed by sample size calculations, with a focus on economic evaluations of these technologies are needed before a case can be made for their widespread adoption in healthcare settings. This is an emerging field, and likely to influence the future of self-directed rehabilitation."*

3. Methods:
I wonder whether additional search terms, including IMUs or accelerometers AND activity or walking, might have broadened the studies found.

Authors' Response: Our search strategy was formulated with the aid of a medical librarian. The inclusion of IMUs and accelerometer broadened the search results significantly and made articles less refined and relevant to consumer grade wearables. We believe the terms chosen such as "activity tracker", "activity sensor" and "wearable device" better encompass consumer grade wearables. Moreover, including terms such as activity and walking also broadens the search results beyond our inclusion criteria, which focuses on rehabilitation and physiotherapy, not general activity. The addition of ("IMUs" OR "Accelerometer") AND ("activity" OR "walking") led to more than 30,000 additional articles, which would make the review too broad and unwieldy.

4. Discussion:
Please include more information about the use of tracking devices on the wrist versus the ankle versus a pocketed smartphone for step counts and distance walked. One investigator in one of the papers said inaccuracy at slow speeds was likely and one study use an ankle device. There is considerable evidence that only ankle sensors are reliable in detecting stepping at <0.5 m/s and that an algorithm must be created to enable recognition of slow walking.

Authors' Response: We comment on the heterogeneity of the wearables used within the discussion as a limitation and acknowledge that there are different devices and methods used in the chosen studies. The majority of consumer grade wearables are worn on the wrist, hence we

did not explore the differences between ankle sensors and wrist sensors in depth. The aim of this review was to explore consumer grade wearables, in the form they are available in the public domain. We also comment that the majority of studies used standard activity outcomes such as step-count as opposed to derived outcome measures, such as that in the study by Fusari et al. which used an algorithm to detect purposeful upper limb movement only, and how there is scope for studies to explore specific derived-outcome measures that are condition or discipline specific. (pages 31-32).

5. Reliability of step counts seems to have been the only aim of the Klassen and the Katzen Fitbit studies. Indeed, it is unclear why these two studies were chosen for inclusion in your review, since, while the Fitbit accurately measured steps taken during rehabilitation of stroke, no assessment was made relevant to the value of the device for activity monitoring of rehabilitation or outcomes.

Authors' Response: Both studies by Klassen and Katzen were directly relevant to the aims of this study and met the inclusion criteria. Both used consumer grade wearables (FitBit). Klassen et al. assessed the accuracy of consumer based physical activity monitor during stroke patient rehabilitation and Katzen et al. aimed to determine stroke patient adherence with wearing a consumer-grade activity tracker, assess its accuracy and evaluate patient experience.

6. Discuss differences between statistically significant changes, e.g., in step counts or distance walked or upper extremity movements and clinically meaningful changes within the reviewed studies.

Did any of the studies assess walking speed based on the devices used? If so, what might be the value and limitations of this measure?

The STARFISH study is an example of uncertainty about which intervention was most valuable or whether all the interventions that included step monitoring may be necessary. Here, social support may have been as important as the bubbling fish for the experimental group. The same holds for the two quasi-experimental cancer studies – was feedback from sensors a critical component of improvements or was the rehabilitation and added support from the research group sufficient? These trials would not appear to add experimental support to your conclusions about the value of activity sensing.

In the Lawrie study, aside from other design problems, the difference of 10% more than baseline activity may not be clinically important. Any outcome for an RCT, of course, ought to be considered for statistical significance in its outcomes and whether differences represent a clinically important change. As in many other chosen papers, such issues limit your ability to draw conclusions about the value of activity sensing.

Regarding your methods to detect bias, please discuss the examples above that may contribute to actual bias in how you rated the studies. It is not always clear how the sensor data was related to any change in outcomes, especially given that most of the uncontrolled studies seem to have included multiple interventions – physiotherapy, inherent social support and personalized care, motivational incentives associated with participation in a study aimed at increasing activity, etc.

Authors' Response: We acknowledge that a limitation of this review is in the inclusion of non-randomised quasi-experimental and observational studies, which are low in the evidence hierarchy. Specifically, in the revised R1 manuscript (page 33), we state "*Finally, there are limitations within the level of evidence included in this review. Twelve studies were level III or IV quasi-experimental or observational studies, and six were level II RCTs. All six RCTs had some concern for bias in at least one domain, and of the non-randomised experimental and*

observational studies, one had serious risk of bias in the selection of participants domain". We also comment that the majority of the studies included low participant numbers. In this revised R1 manuscript, we have used GRADE to assess the certainty of evidence and have allocated low to very low certainty for all the non-randomised studies, to reflect this.

7. Conclusion:
You state: "The current review clearly demonstrates many discipline-wide potential advantages of mHealth and the use of wearables in patient rehabilitation, at the least when used in conjunction with traditional physiotherapy, and at best as feasible cost-effective alternatives."
Although I too am a fan of mHealth and activity sensing for specific purposes, your presentation leaves this reader less than satisfied that the chosen studies and your methods of evaluation adequately support this conclusion. A bit later, you state that wearables require further study as feasible cost-effective alternatives.

Authors' Response: In the revised R1 manuscript, we acknowledge that the majority of included studies are of low evidence certainty. We have revised and toned down the conclusion statement at the end of the discussion section (pages 33-34) to be more balanced and state *"better designed studies, and larger RCTs appropriately informed by sample size calculations, with a focus on economic evaluations of these technologies are needed before a case can be made for their widespread adoption in healthcare settings"*.

8. In the final paragraph, you state that activity sensors can serve as an adjunct to traditional physiotherapy. What do you mean and state how, based on your chosen papers.
You also state that, at best, commercial wearables can be an alternative for self-directed rehabilitation. What do you mean and state how, based on your chosen papers.
Or is this conclusion meant to convey only an aspiration for acute rehabilitation wearables?

Authors' Response: For the vast majority of studies within this review, authors have demonstrated that consumer grade wearables facilitate rehabilitation, at the least as an adjunct to traditional physiotherapy, as an example by providing wearable driven feedback and increasing activity levels. This is summarised in Table 3 (18-19).

Two orthopaedic specific RCTs highlighted that wearables are non-inferior to traditional physiotherapy (Crawford et al. and Tripuraneni et al.) and that wearable driven self-directed rehabilitation is a potential alternative to traditional physiotherapy. In the revised R1 manuscript, we highlight this in the abstract by stating *"Two RCTs in orthopaedics demonstrated non-inferiority of wearable self-directed rehabilitation compared to traditional physiotherapy, highlighting the potential of wearables as alternatives to traditional physiotherapy"*. We also present this in the results section (pages 20-21) and the discussion (page 31), stating *"...two RCTs demonstrating non-inferiority of wearables when compared to traditional physiotherapy. This has significant clinical impact when resources are limited, for example due to lack of physiotherapists, where wearables have the potential to drive self-directed rehabilitation."*.

Finally, we would like to thank you for the thoroughness of your review.

VERSION 2 - REVIEW

**Reviewer**	**1**
**Name**	**Zadro, Joshua**

Affiliation **University of Sydney, Institute for Musculoskeletal Health, School of Public Health**

Date **04-Sep-2024**

COI **None**

The authors have substantially improved the manuscript and I think it will make a great addition to the literature.

Reviewer **3**

Name **Dobkin, Bruce**

Affiliation **UCLA, Neuroscience**

Date **07-Sep-2024**

COI **none**

The authors have responded to the critiques to make the submission more thorough.

Here are several recommendations to help clarify the results and discussion for readers who are less familiar with the many potential outcome measures that can be obtained from commercially available sensors.

In the Abstract, you state:

"comprehensive search of all randomised trials and

observational studies utilising consumer grade wearables for rehabilitation in

healthcare ..." The design, however, was to assess wearables during "acute rehabilitation for non-chronic conditions." Please restate this in the Conclusion and in the first of the listed Strengths and Limitations Of This Study.

In the Abstract and Results, please state the most common types of physical activity that were recorded to classify the outcomes listed in the Tables with greater precision, such as step counts. Physical activity alone is vague.

VERSION 2 - AUTHOR RESPONSE

Reviewer: 1
Mr. Joshua Zadro, University of Sydney
Comments to the Author:

The authors have substantially improved the manuscript and I think it will make a great addition to the literature.

Author Response:

Many thanks for your review and feedback, and for confirming that this manuscript will make a valuable addition to the literature.

Reviewer: 3
Dr. Bruce Dobkin, UCLA
Comments to the Author:

The authors have responded to the critiques to make the submission more thorough. Here are several recommendations to help clarify the results and discussion for readers who are less familiar with the many potential outcome measures that can be obtained from commercially available sensors.

In the Abstract, you state:
"comprehensive search of all randomised trials and
observational studies utilising consumer grade wearables for rehabilitation in healthcare ..." The design, however, was to assess wearables during "acute rehabilitation for non-chronic conditions." Please restate this in the Conclusion and in the first of the listed Strengths and Limitations Of This Study.

Author Response:

We have updated the first bullet point in the Strengths and Limitations section to read: "This systematic review used a comprehensive search of all randomised trials and observational studies utilising consumer grade wearables for rehabilitation of *non-chronic conditions* in healthcare, which has not been undertaken in the literature previously."

We have also included "non-chronic conditions" in the conclusion section of the abstract.

In the Abstract and Results, please state the most common types of physical activity that were recorded to classify the outcomes listed in the Tables with greater precision, such as step counts. Physical activity alone is vague.

Author Response:

Within the results section of the Abstract, we have now included reference to step-count being the most utilised measure of physical activity, stating: "Step-count was the commonest measure of physical activity."

We have also updated the Results section, pages 14-15 under the section *Wearable Technologies, Operating Systems and Outcome Measures*, and included reference to step-count, stating: "Step-count was the most commonly utilised outcome measure of physical activity."

We thank you again for your thorough review and feedback.
